# Enhanced SOX2 expression in retinoblastoma tissues and peripheral blood is associated with the clinicopathological characteristics of the disease

**DOI:** 10.3892/ol.2015.2857

**Published:** 2015-01-07

**Authors:** BODING TONG, JIEXI ZENG, YUJIE WU, WEI XIONG

**Affiliations:** Department of Ophthalmology and Eye Research Center, The Second Xiangya Hospital, Central South University, Changsha, Hunan 410011, P.R. China

**Keywords:** retinoblastoma, SOX2, immunohistochemistry staining

## Abstract

The present study aimed to investigate the association between the expression of sex-determining region Y box 2 (SOX2) in retinoblastoma (Rb) tissues and peripheral blood, and the clinicopathological characteristics of Rb. The expression of SOX2 in Rb tissues was detected by immunohistochemical staining and western blot analysis. SOX2 expression in the peripheral blood of children with Rb was determined using quantitative real-time polymerase chain reaction. The correlation between SOX2 expression and the clinicopathological characteristics of Rb was analyzed using χ^2^ tests. The positive rate of SOX2 in Rb tissues was 82.2%, while the expression of SOX2 in the control group tissues was negative. Western blot analysis detected a higher expression of SOX2 in the Rb tissues than in the control group tissues. Poorly differentiated Rb tissues exhibited significantly higher levels of SOX2 expression compared with the well-differentiated Rb tissues. SOX2 expression was higher in the peripheral blood of children with Rb than in individuals from the control group. The level of SOX2 expression in the peripheral blood of the poorly differentiated group was higher than that of the well-differentiated group. Enhanced SOX2 expression in Rb tissues and peripheral blood was closely associated with the clinicopathological characteristics of Rb. Therefore, SOX2 may be a novel target biomarker for the clinical diagnosis and treatment of Rb.

## Introduction

Retinoblastoma (Rb) is a common primary intraocular malignancy that affects children. Rb can cause serious damage to the eyes and vision, or may lead to mortality as a result of intracranial and systemic metastases at a later stage (1). Previous studies have identified that multiple genes have an important role in the incidence and development of Rb (2,3). The Rb gene demonstrates good prospects for the early diagnosis, treatment, pathological classification and prognosis of Rb (4). Therefore, the search for novel genes, which are associated with Rb, has become a topic of interest in recent years (5,6). The sex-determining region Y box 2 (SOX2) is an embryonic stem cell gene, which has a key role in embryonic tissue development (7). An abnormal expression of SOX2 has been identified in lung, stomach, liver and breast cancers, and in other tumors, which suggests that SOX2 may function in tumor development, invasion and metastasis (8–10). At present, the role of SOX2 expression in Rb, and its underlying mechanisms, are unclear. The present study aimed to analyze the gene and protein expression of SOX2 in the Rb tissues of 45 children, and in the peripheral blood of 15 children with Rb, and also identify any association between SOX2 expression and the clinicopathological features of the disease.

## Materials and methods

### Tissue samples

Tissue samples were collected from 45 children with Rb (28 male and 17 female; aged between 1 month and 108 months, with a mean age of 32 months) who had undergone treatment at the The Second Xiangya Hospital (Changsha, China), between December 2010 and December 2013. Of these patients, 32 had unilateral Rb and 13 had bilateral Rb. The children were grouped according to the International Intraocular Retinoblastoma Classification (10) as follows: i) N0 stage, no invasion of the optic nerve by the tumors; ii) N1 stage, tumor invasion of the optic nerve head that did not exceed the sieve; iii) N2 stage, tumor penetrated through the sieve, but no tumor cell invasion of the optic nerve stump; and iv) N3 stage, tumor cells at optic nerve stump. The tissues were divided into a well-differentiated group and a poorly differentiated group, depending upon whether the tumor cells were arranged into a Flexner-Winterstein rosette. In the present study, six cases were at N0 stage, five cases were at N1 stage, 16 cases were at N2 stage and 18 cases were at N3 stage. In total, 31 samples belonged to the well-differentiated group and 14 samples belonged to poorly differentiated group. In addition, 15 pieces of normal retinal tissue, which had surrounded the Rb tumors, were collected to represent the control group. Written informed consents were obtained from the family members of the patients, and the study was approved by the Ethics Review Board of Central South University, Changsha, China.

### Reagents

The serum RNA extraction reagent TRIzol LS and the total RNA extraction reagent TRIzol were purchased from Invitrogen (Carlsbad, CA, USA). The rabbit anti-human SOX2 polyclonal antibodies were purchased from Santa Cruz Biotechnology, Inc. (Santa Cruz, CA, USA). The reverse transcription system was purchased from Takara Bio, Inc. (Shiga, Japan). The streptavidin-peroxidase immunohistochemical kits were purchased from Beijing Zhongshan Co. (Beijing, China). The quantitative reverse transcription polymerase chain reaction (qRT-PCR) kit for mRNA was purchased from Kapa Biosystems, Inc. (Wilmington, MA, USA).

### Immunohistochemistry

The streptavidin-peroxidase two-step method was used for the immunohistochemical staining. A portion of the tumor tissues was fixed in 10% formaldehyde, embedded in paraffin, sliced and then soaked in xylene diluent for dewaxing. Subsequent to blocking non-specific binding with horse serum, the polyclonal rabbit anti-human SOX2 antibody (dilution, 1:200; cat. no. ab171380) was added, followed by a 30-min incubation with biotin-labeled polyclonal goat anti-mouse IgG (dilution, 1:3,000; cat. no ab6789) and polyclonal goat anti-rabit IgG (dilution, 1:2,000; cat. no. ab6721) secondary antibodies (Abcam, Cambridge, MA, USA) at 37°C. After washing three times with phosphate-buffered saline, the streptavidin-peroxidase complexes were added to the samples for 30 min. Next, diaminobenzidine staining was performed. After washing with phosphate-buffered saline three times, the mixture was mildly stained with hematoxylin and eosin, differentiated with hydrochloric acid, washed with tap water, dehydrated with graded alcohol and then mounted with neutral gum.

### Microscopy

Each slice was observed under an Olympus BX53 optical microscope (Olympus Corporation, Tokyo, Japan) (magnification, ×400). Brown or sepia granular staining of the cytoplasm or cellular membrane indicated positive cells. In total, five high-power fields were randomly selected for the tumor cell count. The percentage of positive cells was represented by the ratio of stained tumor cells to the total number of tumor cells in the field. The average percentage of positive cells was calculated from the five fields. The final score was calculated as follows: Final score = degree of staining × percentage of positive cells in each field. The final scores were presented as follows: 0–1, negative; 2–3, weak positive; 4–6, positive; >6, strong positive.

### Western blotting

In order to extract the total proteins, Rb tissues were lysed with radio-immunoprecipitation assay protein (Beyotime Institute of Biotechnology, Haimen, China). Next, SDS-PAGE was performed for 2 h. The proteins were then transferred to a polyvinylidene difluoride membrane, and blocked with 5% skimmed milk for 1 h at room temperature. Next, primary antibodies against the target protein, SOX2 (dilution, 1:1,000), and the internal reference protein, GAPDH (polyclonal rabbit anti-human antibody; dilution, 1:3,000; cat. no. ab9485; Abcam), were added proportionally. The horseradish peroxidase-labeled secondary antibodies for SOX2 (goat anti-mouse; dilution, 1:3,000) and GAPDH (goat anti-rabbit; dilution, 1:2,000) were then added, followed by incubation at room temperature for 1 h. The membrane was then washed with phosphate-buffered saline with Tween 20 three times for 15 min each time, and developed with electrochemiluminescence liquid.

### RT-qPCR

Overall, 6 μl total RNA was used for the reverse transcription reaction, and the expression of SOX2 in the peripheral blood was determined by RT-qPCR. For reverse transcription, the reaction conditions were as follows: 42°C for 45 min. The reaction system consisted of 10 μl qRT-PCR-Mix, 0.5 μl upstream primer, 0.5 μl downstream primer, 1 μl cDNA and 8 μl ddH_2_O. The reaction conditions for PCR were as follows: pre-denaturation at 94°C for 5 min, followed by 40 cycles of 94°C for 30 sec, 60°C for 30 sec and 72°C for 20 sec, and a final extension step at 72°C for 5 min. Three parallel wells were set up for each sample. The internal reference gene was GAPDH, and the associated primers are listed in Table I. The 2^−ΔΔCT^ method was used to calculate the relative expression level of SOX2.

### Statistical analysis

Data was analyzed using SPSS version 16.0 statistical software (SPSS, Inc., Chicago, IL, USA). The data are expressed as the mean ± standard deviation. Group t-test was used for the comparison of SOX2 expression between groups in the peripheral blood of Rb children. The χ^2^ test was used to analyze the association of SOX2 protein expression with the clinicopathological features of Rb. A value of P<0.05 was used to indicate a statistically significant difference.

## Results

### SOX2 expression in Rb tissues is dependent upon the degree of Rb differentiation and optic nerve invasion

**Figure 1:**
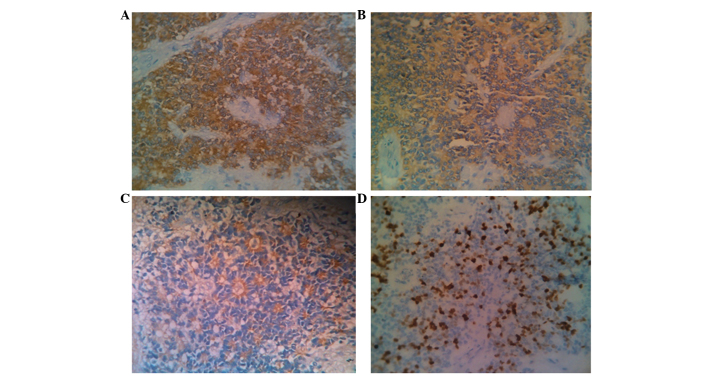
Immunohistochemical analysis revealing the expression of sex-determining region Y box 2 (SOX2) protein in retinoblastoma tissues (magnification, ×10). (A) Strong positive expression of SOX2. (B) Positive expression of SOX2. (C) Weak positive expression of SOX2 in the cytoplasm. (D) Positive expression of SOX2 in the nucleus.

In order to analyze the expression of SOX2 in Rb tissues, immunohistochemical staining was performed. The results revealed that SOX2 was expressed either in the cytoplasm or the cell membrane of Rb cells, or in both (Fig. 1). SOX2 expression in Rb tissues was positive, and included 10 weak positive cases (22.2%), 14 positive cases (31.1%) and 21 strong positive cases (46.7%). By contrast, SOX2 expression in the control group was negative. The comparison of SOX2 expression between groups of varying Rb differentiation revealed that the expression was strong positive in the 14 cases of poorly differentiated Rb. However, in the well-differentiated group, there were seven cases of strong positive expression, 14 cases of positive expression, and 10 cases of weak positive expression. The comparison of SOX2 expression between groups of different Rb stages revealed that there were nine cases of weak positive SOX2 expression, and two cases of positive SOX2 expression in the N0–N1 stage tissues; four cases of strong positive SOX2 expression, 11 cases of positive SOX2 expression and one case of weak positive SOX2 expression in the N2 stage tissues; and 17 cases of strong positive SOX2 expression and one case of positive SOX2 expression in the N3 stage tissues. The comparison between genders revealed that there were no statistically significant differences between the SOX2 positive expression rates (P<0.05) (Table II). These data suggest that SOX2 expression in Rb tissues is dependent upon the degree of Rb differentiation and optic nerve invasion.

### SOX2 protein expression is correlated with the development and invasion of Rb

**Figure 2:**
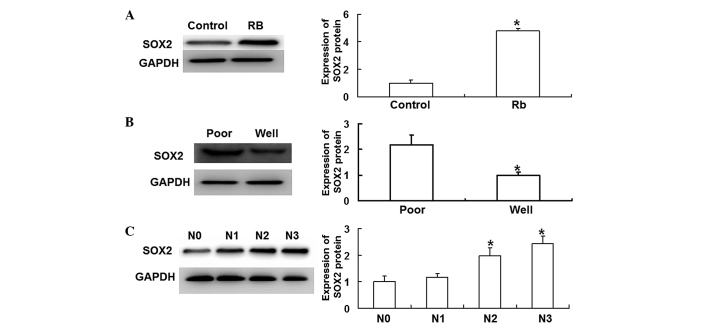
Sex-determining region Y box 2 (SOX2) protein expression in retinoblastoma (Rb) tissues, as detected by western blot analysis. The quantification of western blots were plotted into histograms. (A) SOX2 protein expression in Rb and control tissues. ^*^P<0.05, compared with the control group. (B) SOX2 protein expression in the poorly and well-differentiated groups. ^*^P<0.05, compared with the poorly differentiated group. (C) SOX2 protein expression in Rb tissues at stages N0, N1, N2 and N3. ^*^P<0.05, compared with the N0 group. All data are presented as the mean ± standard error of the mean.

Western blot analysis was performed in order to measure the expression of SOX2 protein within the Rb tissues. The results revealed that SOX2 protein expression was significantly higher in the Rb tissues than in the control group tissues (P<0.05) (Fig. 2A). In addition, SOX2 protein expression was significantly higher in the poorly differentiated group than in the well-differentiated group (P<0.05) (Fig. 2B). Furthermore, SOX2 protein expression increased as the optic nerve invasion progressed from stage N0 to N3, with the expression significantly higher at the N2 and N3 stages than the N0 stage (P<0.05) (Fig. 2C). These results suggest that SOX2 protein expression is correlated with the development and invasion of Rb.

### SOX2 gene is highly expressed in the peripheral blood of Rb children, with expression in the poorly differentiated group higher than that in well-differentiated group

**Figure 3:**
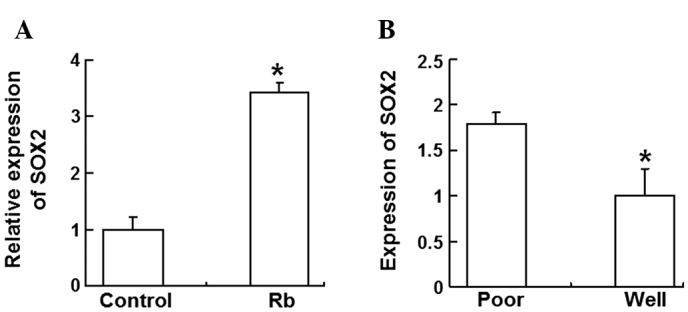
Sex-determining region Y box 2 (SOX2) gene expression in the peripheral blood of children with retinoblastoma (Rb). The quantification of the quantitative reverse transcription polymerase chain reactions were plotted into histograms. (A) Relative expression of SOX2 mRNA in the peripheral blood of control individuals and Rb patients. ^*^P<0.05, compared with the control group. (B) Expression of SOX2 mRNA in the peripheral blood of the poorly and well-differentiated groups. ^*^P<0.05, compared with the poorly differentiated group. All data are presented as the mean ± standard error of the mean.

In order to determine the SOX2 gene expression in the peripheral blood of children with Rb, RT-qPCR was performed. In total, SOX2 gene expression was detected in 15 Rb children, including five poorly differentiated cases and eight well-differentiated cases. The quantitative results demonstrated that SOX2 gene expression was significantly higher in the peripheral blood of Rb children than in individuals from the control group (P<0.05) (Fig. 3A). In addition, SOX2 gene expression in the poorly differentiated group was higher than that observed in the well-differentiated group (P<0.05) (Fig. 3B). These data indicate that the SOX2 gene was highly expressed in the peripheral blood of children with Rb, with expression higher in the poorly differentiated group compared with the well-differentiated group.

## Discussion

In recent years, genes have been discovered that are closely associated with the development of Rb (12,13). Previous studies have demonstrated that Rb gene mutations and deletions occur in the majority children with Rb (14,15). Furthermore, *in vitro* experiments have indicated that the Rb gene has important roles in the incidence, metastasis, apoptosis and other aspects of Rb. Vascular endothelial growth factor has been demonstrated to promote Rb tumor angiogenesis (16). The melanoma differentiation associated gene-7 has been shown to selectively induce the apoptosis and inhibit the growth of Rb tumor cells (17). The identification of these novel biomarkers has led studies to investigate the molecular mechanism of Rb, and has provided an alternative approach for clinical diagnosis and treatment.

The SOX2 gene, a member of the SOX gene family, is an important transcription factor that regulates embryotic development and cell differentiation, and which functions in the embryotic development of the brain, nerves, lens and other tissue structures. The abnormal expression of SOX2 can often lead to cellular and tissue differentiation in developmental disorders (18,19). Therefore, the function of the SOX2 gene in abnormally differentiated tumor tissues has become of particular interest. Previous studies have demonstrated that the expression of SOX2 is increased in lung, liver and stomach cancers, and in other tumor tissues, and that it is positively correlated with the clinicopathological stages and degrees of differentiation of Rb (20,21). *In vitro* experiments have confirmed that SOX2 functions in the malignant biological behaviors of a variety of tumor cells (22,23). In the present study, immunohistochemistry, western blotting and RT-qPCR were performed in order to detect the expression of SOX2 in Rb tissues and peripheral blood. In addition, the correlations between SOX2 expression and the degree of differentiation and clinical stage of Rb were preliminarily analyzed using the clinicopathological data. It has been reported that SOX-2 expression is elevated in Rb tissues (14,24–27). For example, Wadhwa *et al* (24) observed that SOX2 is expressed in the inner retina and the ganglion cells of human RB tumors. Zhang et al (25) identified Sox2 as one of the upregulated genes in Rb tissues using a chromatin immunoprecipitation-on-chip analysis. Our results were consistent with these previous reports.

The results of the present study indicate that the SOX2 protein, as a key transcription factor in the embryotic development and tissue cell differentiation, has an important role in the incidence and development of Rb. Due to the diversity of the downstream target genes that can be regulated by SOX2, the specific molecular mechanism of SOX2 requires further study. The peripheral blood results suggested that SOX2 gene expression may be clinically useful for the early diagnosis and treatment of children with Rb.

To summarize, the SOX2 gene was highly expressed in Rb tissues and in the peripheral blood. Furthermore, its expression increased with the progression of clinical stage and with the lowering of the degree of differentiation. The present study indicates that SOX2 has an important role in the incidence and development of Rb. However, the downstream molecular mechanism requires further study in order to provide a theoretical basis for the clinical diagnosis and treatment of Rb.

## Figures and Tables

**Table I:** Primers for quantitative reverse transcription polymerase chain reaction.

Primer	Sequence (5′ to 3′)
SOX2, forward	GAGAGTGTTTGCAAAAGGGGG
SOX2, reverse	GCTTCTCCGTCTCCGACAAA
GAPDH, forward	CTCTGCTCCTCCTGTTCGAC
GAPDH, reverse	GCGCCCAATACGACCAAATC

SOX2, sex-determining region Y box 2.

**Table II:** Correlation of sex-determining region Y box 2 (SOX2) expression and clinicopathological features of retinoblastoma.

Variables	n	Percentage of positive SOX2 expression	χ^2^	P-value
Gender				
Male	28	100		
Female	17	100		
Optic nerve invasion			29.152	0.000
N0–1	11	0^a^		
N2	16	25^a^		
N3	18	94^a^		
Differentiation			23.226	0.000
Well	31	23^a^		
Poor	14	100^a^		

a Percentage of strong positive SOX2 expression.
